# Meta-Research in Neuroscience: An Urgent Call to Strengthen the Reliability and Translation of Knowledge into Evidence-Based Neurological Practice

**DOI:** 10.3390/jcm14238552

**Published:** 2025-12-02

**Authors:** Ivan David Lozada-Martinez, Luis Miguel Delgado Arias, Diana Marcela Perea Rojas, Indiana Luz Rojas Torres

**Affiliations:** 1 Biomedical Scientometrics and Evidence-Based Research Unit, Department of Health Sciences, Universidad de la Costa, Barranquilla 080002, Colombia; 2 Clínica Iberoamérica, Barranquilla 080001, Colombia; 3 Facultad de Ciencias para la Salud, Universidad de Manizales, Manizales 170001, Colombia; luismigueldel1@hotmail.com; 4 Faculty of Health Sciences, Universidad Simón Bolívar, Barranquilla 080003, Colombia; dianaperea1822@gmail.com; 5 Facultad de Ciencias de la Salud, Centro de Investigaciones en Ciencias de la Vida, Universidad Simón Bolívar, Barranquilla 080003, Colombia

**Keywords:** meta-research, neurosciences, evidence gaps, evidence-based practice, research design

## Abstract

Despite the exponential expansion of neuroscience over recent decades, the field has rarely examined the rigor, transparency, and reproducibility of its own evidence base (research on research). Through a brief systematic exploration of the Scopus database, we identified more than 370,000 neuroscience articles, yet only 15 explicitly addressed meta-research questions, representing a mere 0.004% of the total literature. These few studies were concentrated in high-income countries and limited mainly to neuroimaging and methodological reporting, leaving major subfields such as neuropharmacology, neuropathology, and neuroendocrinology virtually unexplored. This striking imbalance reveals a systemic absence of evidence self-assessment and highlights how neuroscience has advanced without adequate reflection on the validity and translatability of its findings. The lack of meta-research weakens the reliability of neuroscientific evidence, slows the development of shared reporting standards, and risks compromising its translation into evidence-based neurological practice. Strengthening global collaboration, fostering reflexivity, and integrating meta-research into neuroscience are urgent steps toward ensuring that the knowledge generated in laboratories and trials truly supports trustworthy, reproducible, and clinically meaningful clinical healthcare in neurological sciences.

## 1. The Role of Meta-Research in Advancing Neuroscience

In recent decades, neuroscience has become one of the most rapidly expanding areas of medicine [1]. Every year, thousands of studies seek to explain how the brain works, how it fails in disease, and how it might be repaired [2]. This collective effort has produced an extraordinary amount of knowledge, yet much less attention has been given to examining how that knowledge is generated and whether it can truly be trusted [1,2,3].

Meta-research (the study of how research itself is performed) provides a framework for asking those questions [4,5]. It explores whether studies follow rigorous methods, report their findings transparently, and produce results that can be reproduced and applied [4,5]. Rather than generating new data, meta-research evaluates the quality of the evidence that already exists [4,5]. This approach is essential because scientific progress depends not only on producing results, but also on understanding how those results come to be [4,5,6].

From the perspective of evidence-based medicine, meta-research has helped expose problems that often remain hidden: studies with small samples or weak designs, selective reporting of favorable outcomes, and the repetition of low-quality reviews [7]. These issues can quietly distort the conclusions that guide clinical decisions [7]. In fields such as oncology and psychology, acknowledging these limitations has led to reforms in how research is planned, published, and assessed, making their evidence more reliable and clinically useful [7].

For neuroscience, this kind of reflection is urgently needed. The field produces enormous and diverse bodies of data, from laboratory models to clinical trials, all of which shape how neurological care is practiced [1,2,3]. Without systematic evaluation of their quality and reproducibility, these efforts risk generating volume rather than certainty [7]. Strengthening meta-research within neuroscience is therefore a necessary step to ensure that its discoveries truly contribute to sound, evidence-based clinical practice.

It is important to acknowledge that certain subfields, particularly neuroimaging, have benefited from major initiatives aimed at improving transparency and methodological rigor, including COBIDAS guidelines [8], the Brain Imaging Data Structure (BIDS) [9], OpenNeuro [10], and the ReproNim framework [11]. These initiatives have substantially strengthened data-sharing practices and analytical standardization [8,9,10,11]. However, these efforts primarily represent open science and methodological infrastructure rather than formal meta-research, which focuses on systematically evaluating research practices, bias, reporting quality, and reproducibility across the literature. When considering the field as a whole, we suspect that formal meta-research output in neuroscience remains extremely scarce.

This study aimed to identify and quantify the presence of meta-research within the field of neuroscience, highlighting the magnitude of its methodological gap and discussing its implications for the reliability and translation of neuroscientific knowledge into evidence-based clinical practice.

## 2. Exploring the Evidence Gap: Mapping Meta-Research Within Neuroscience

To explore the landscape to which neurosciences research has been accompanied by meta-research approaches, we conducted a structured search in Scopus (at 20 September 2025). The strategy combined controlled descriptors and synonyms related to neuroscience (MeSH Unique ID: D009488) (e.g., neuroanatomy, neurophysiology, neurochemistry, cognitive neuroscience, others) with those referring to meta-research (MeSH Unique ID: D000098346) or meta-science. Conference papers, book chapters, and proceedings were excluded; only peer-reviewed publications published in regular issues of scientific journals were considered, excluding articles in press. This methodology has been previously used for this same approach and similar objectives [4,5].

The exact strategy used was: (TITLE-ABS-KEY(Neuroscience*) OR TITLE-ABS-KEY(Cognitive Neuroscience*) OR TITLE-ABS-KEY(Social Neuroscience*) OR TITLE-ABS-KEY(Neuroanatom*) OR TITLE-ABS-KEY(Neurobiology) OR TITLE-ABS-KEY(Neurochemistr*) OR TITLE-ABS-KEY(Neuroendocrinology) OR TITLE-ABS-KEY(Neuropatholog*) OR TITLE-ABS-KEY(Neuropharmacolog*) OR TITLE-ABS-KEY(Neurophysiology)) AND (TITLE-ABS-KEY(Meta-Research) OR TITLE-ABS-KEY(“Meta Research”) OR TITLE-ABS-KEY(Meta-science) OR TITLE-ABS-KEY(“Meta science”)). No date restrictions were applied to capture the full historical trajectory of neuroscience meta-research.

Although neuroscience publications are dispersed across multiple indexing platforms (e.g., PubMed, Web of Science, Embase, PsycINFO), Scopus was selected as the primary data source because it provides the broadest journal coverage in neuroscience and the richest bibliometric metadata (including author identifiers, complete keyword corpora, institutional affiliations, and country-level classifications). During the exploratory phase, complementary searches were conducted in PubMed, Web of Science, and PsycINFO, but these platforms yielded substantially fewer records and a high proportion of duplicates, and did not allow extraction of the metadata required for co-authorship, geographic, and keyword-based analyses. Therefore, Scopus offered the most consistent, reproducible, and analytically suitable dataset for the objectives of this study [4,5].

Bibliometric data (authors, countries, affiliations, keywords, and year of publication) were exported for descriptive analysis. We examined co-authorship networks, geographic distribution, and economic grouping of countries (based on World Bank classification [12]), as well as temporal trends of publication.

All analyses were descriptive and exploratory. Bibliometric metadata were processed in Python (v.3.14) using pandas (v.2.3.3) for data structuring and network (v.3.6) for co-authorship network construction [13]. Visualizations were produced using matplotlib (v.3.10.7) and seaborn (v.0.13.2). Geographic distributions were computed through frequency counts and mapped using geopandas (v.1.1.1). Keyword analyses were performed by generating a co-occurrence matrix of author keywords; terms appearing fewer than two times were excluded to improve signal-to-noise ratio. Clustering was based on a frequency-weighted co-occurrence model, and thematic groups were identified using the Louvain community detection algorithm implemented in the python-louvain package (v.0.16). For validation, parallel analyses were performed in RStudio (v.4.5.2) using igraph (v.2.2.1) and ggplot2 (v.4.0.1) [14]. The resulting clusters were therefore derived from the natural community structure of the co-occurrence network, rather than from inferential or predictive models, consistent with the exploratory aims of the study.

**Figure 1:**
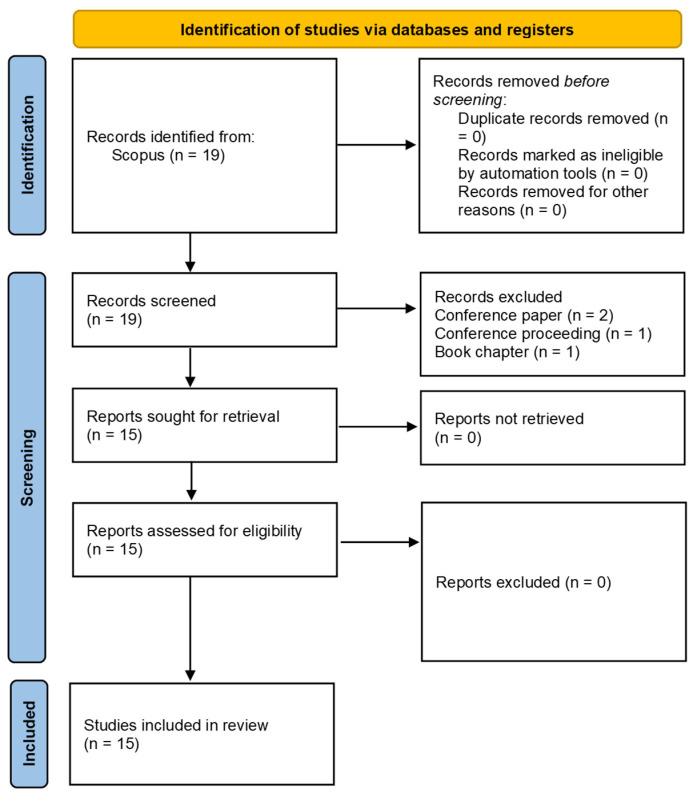
Flowchart of selected documents related to neuroscience and meta-research.

A total of 378,072 neuroscience articles were found in Scopus, while 974 publications were only to meta-research issues regardless of the discipline of knowledge to which it was applied. The intersection of both domains yielded only 19 records, of which 15 articles were included after exclusions (Figure 1). This corresponded to 0.004% of the total neuroscience literature, evidencing a marked gap between the volume of research and the absence of methodological and research self-assessment. The earliest article addressing meta-research in neuroscience was published in 2017, and the most recent in 2024, covering a period of just seven years. During this interval, an average of two papers per year were published, contrasting with the more than 20,000 neuroscience papers per year indexed in Scopus over the same period.

**Figure 2:**
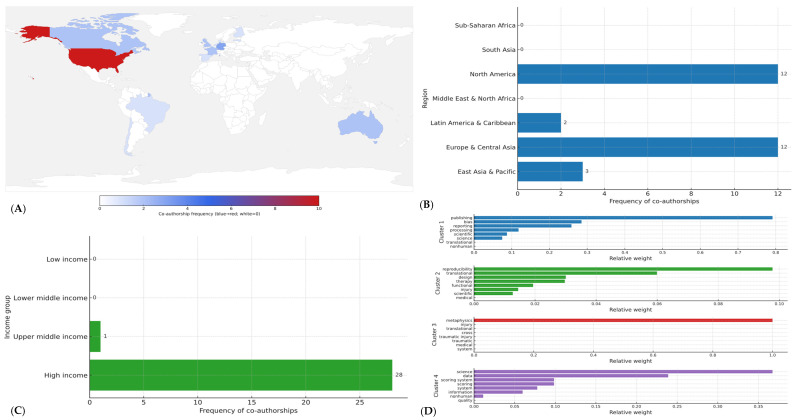
Global patterns and thematic clusters of meta-research in neurosciences. (**A**) Global distribution of co-authorship frequencies in meta-research and neurosciences. (**B**) Contributions by World Bank geographic regions. (**C**) Contributions by World Bank income groups. (**D**) Main thematic lines in meta-research applied to neurosciences (clusters of key terms). This figure displays four thematic clusters derived from the analysis of article titles and keywords. Each horizontal bar represents a representative term within the cluster, and its length indicates the relative weight (statistical importance in the clustering model). In practical terms, a higher weight means that the term is more frequent and distinctive in that set of articles, thus better characterizing the thematic line. The clusters therefore highlight the areas of neurosciences where meta-research publications have been concentrated, as well as those domains where gaps and opportunities for future studies remain.

In total, 29 co-authorships across 14 countries were identified (Figure 2A). By World Bank region, contributions were concentrated in North America (41.4%, *n* = 12) and Europe & Central Asia (41.4%, *n* = 12), with smaller shares from East Asia & Pacific (10.3%, *n* = 3) and Latin America & Caribbean (6.9%, *n* = 2) (Figure 2B); no co-authorships were observed from South Asia, Middle East & North Africa, or Sub-Saharan Africa (Figure 2A).

By income level, high-income economies accounted for 96.6% (*n* = 28) of all co-authorships, upper-middle-income settings for 3.4% (*n* = 1), and no contributions were registered from lower-middle or low-income economies (Figure 2C). At the country level, the United States contributed 34.5% (*n* = 10) of co-authorships; Germany contributed 10.3% (*n* = 3); and Australia, Canada, France, and the United Kingdom contributed 6.9% each (*n* = 2) (Figure 2A). The remaining eight countries contributed single co-authorships each (*n* = 1). Overall participation appeared fragmented: 12/14 countries (85.7%) had ≤2 co-authorships each, yet collectively accounted for 55.2% (*n* = 16/29) of all co-authorships, indicating breadth without depth and a collaboration base concentrated in affluent regions.

Thematic clustering of titles and keywords identified four main lines of inquiry (Figure 2D): (1) Reproducibility and reporting standards in neuroimaging studies (functional magnetic resonance imaging, electroencephalogram), representing approximately 30% of the articles); (2) Methodological biases in clinical neuroscience trials, observed in 25% of the set; (3) Open science practices and data sharing in neuroscience, accounting for another 25%; and (4) Cross-disciplinary perspectives connecting neuroscience with research integrity frameworks, found in the remaining 20% (Figure 2D).

By contrast, major subfields such as neuropharmacology, neuropathology, and neuroendocrinology, which together represent tens of thousands of publications, were absent from the meta-research corpus. These thematic silences highlighted critical knowledge gaps, where the lack of methodological evaluation may compromise both the reliability and the translation of findings into clinical practice [15].

Then, the present findings reveal a critical gap: more than 370,000 neuroscience papers were indexed, yet only 15 explicitly addressed meta-research questions. This striking disproportion reflects a systemic neglect of knowledge self-evaluation. From an epistemological perspective, the absence of meta-research undermines the ability of neuroscience to critically validate its own claims, leaving large areas of knowledge unexamined [16]. This lack of reflexivity may foster accumulation of evidence without adequate scrutiny of its internal coherence [16].

## 3. Implications for Reliability and Evidence-Based Neurological Practice

In medicine, progress depends on more than producing data; it depends on knowing whether that data is reliable, reproducible, and relevant to patient care [17,18]. Meta-research, plays a central role in this process. It helps us look beneath the surface of studies to ask critical questions: Were the methods appropriate? Were the results reported transparently? Can others reproduce the findings? [7,19].

The implications of the present findings should be interpreted in light of well-documented reproducibility concerns across several neuroscience subfields. For example, neuroimaging has faced major challenges in analytical flexibility, inflated false-positive rates, and inconsistent preprocessing pipelines, as demonstrated by large-scale assessments of functional magnetic resonance imaging reproducibility [20]. Preclinical neuroscience has similarly shown high risks of bias related to small sample sizes, lack of blinding, and selective reporting [21]. Electroencephalography and event-related potential research has also been criticized for methodological heterogeneity and insufficient transparency in data processing workflows [20]. In clinical neuroscience, trials for stroke, dementia, and neuropsychiatric disorders frequently exhibit reporting limitations, selective outcome definitions, and difficulties in reproducing early promising results. The scarcity of meta-research that we identified therefore interacts with a broader pattern of methodological vulnerability already described in the literature [4].

In this context, the absence of systematic self-evaluation does not generate concerns by itself; rather, it exacerbates known weaknesses and limits the field’s ability to correct them, thereby increasing the risk that unreliable evidence is translated into clinical decision-making.

When a scientific field grows rapidly, it often focuses on discovery rather than reflection. Thousands of studies explore how the brain works, but few examine how these studies themselves are designed, reported, or validated. This imbalance can lead to what could be called an illusion of progress: an expanding volume of publications that may not necessarily strengthen the foundation of knowledge [19]. Without self-evaluation, errors can multiply quietly, small sample sizes, selective reporting, or analytical biases, that later influence systematic reviews, clinical guidelines, and, ultimately, patient treatment [19].

In evidence-based medicine, every clinical decision should be supported by the best available and most trustworthy evidence [22]. Yet if the studies forming that evidence base are not critically assessed for quality and reproducibility, even well-intentioned decisions can rest on uncertain ground [17]. For example, in neuroimaging research, different analyses of the same brain data have sometimes produced conflicting conclusions about brain function or disease mechanisms [23]. Without meta-research to identify why this happens, clinicians may base interpretations on evidence that looks convincing but is not fully stable or reproducible.

This gap has practical consequences. In neurology, unreliable or poorly validated evidence can affect how diseases are diagnosed, how treatments are prioritized, and how new therapies are tested [24]. The lack of systematic self-assessment means that promising discoveries from laboratories or small clinical trials may fail when applied to real-world patients [25]. The result is slower translation of scientific advances into meaningful health outcomes.

From an epidemiological standpoint, the concentration of contributions in high-income countries and the almost complete absence from low- and middle-income settings reflects persistent global population gap (referring to the mismatch between the global populations who ultimately rely on neuroscientific evidence and the small group of countries where such evidence, and its methodological evaluation, is generated. This disparity limits the applicability of neuroscientific knowledge to diverse population contexts) [8]. Such asymmetry threatens the external validity and representativeness of methodological critiques, perpetuating a view of neuroscience shaped only by the contexts where meta-research is conducted [26].

Methodologically, the small and fragmented co-authorship networks, with more than half of contributing countries producing only one or two collaborations, indicate that no consolidated global community of meta-research in neuroscience yet exists. This fragmentation limits cumulative knowledge, slows the development of shared standards, and weakens the potential for comparative analyses across subfields [27] such as neuropharmacology, neuropathology, or neuroendocrinology, where meta-research is completely absent.

From the lens of implementation science, the scarcity of evidence on reporting practices, reproducibility, and data sharing creates barriers for translating neuroscientific discoveries into clinical and public health impact [28,29]. Without systematic evaluations of bias, transparency, and reproducibility, efforts to implement neuroscience findings into practice risk being ineffective, inefficient, or even harmful [28,29].

Meta-research helps prevent this by bringing medicine back to its scientific roots: observation, verification, and reflection. It complements evidence-based medicine by ensuring that the evidence we use is not just abundant but trustworthy. While evidence-based medicine asks “What does the research show?”, meta-research asks “Can we trust how that research was done?”, and both questions are essential for safe, effective, and ethical clinical practice.

However, these international patterns should be interpreted with caution, as meta-research remains a relatively new discipline and the absolute number of identified studies is small [4]. Consequently, geographic comparisons in this analysis reflect early-stage dynamics rather than mature structural trends. The observed concentration of contributions in high-income countries may therefore represent the initial development of the field rather than stable global disparities and should be viewed as exploratory rather than definitive.

Because the total number of identified meta-research studies was small, conducting subfield-specific analyses or applying sensitivity tests and confidence intervals was not feasible. These methods require sample sizes that allow for variability assessment, which is not present in this early-stage evidence base [30]. Therefore, the observed thematic gaps, such as the absence of retrieved studies in neuropharmacology, neuropathology, or neuroendocrinology, should be interpreted as the absence of identified Scopus-indexed publications under our search strategy, rather than definitive evidence that such research does not exist. These findings highlight the preliminary nature of meta-research activity in neuroscience and reinforce the need for more systematic efforts across subfields.

Building this culture of reflection within neuroscience could transform the field. It would help researchers design more transparent studies, improve reporting standards, and create stronger links between basic discoveries and patient care. Ultimately, integrating meta-research into neuroscience is not an abstract academic exercise, it is a practical step toward making neurology more reliable, equitable, and truly evidence-based [31,32].

## 4. Future Directions and Translational Opportunities

Building a culture of meta-research within neuroscience requires more than recognizing its absence, it demands concrete steps to integrate self-evaluation into the scientific and clinical workflow. To make neuroscience more reliable, reproducible, and clinically meaningful, a practical roadmap can guide the next phase of development:

A. Establishing meta-research frameworks within neuroscience institutions: Universities, hospitals, and research centers could create dedicated meta-research units or observatories that regularly evaluate the quality, transparency, and reproducibility of neuroscientific studies [28]. These teams would not replace traditional research groups but complement them, ensuring that methodological soundness is considered as important as discovery itself [31,32].

B. Embedding methodological audits in clinical and experimental research: Before launching large clinical trials or laboratory projects, teams should perform methodological audits to identify design weaknesses, bias risks, or inconsistencies with previous evidence [15,18]. This practice mirrors the pre-flight checks in aviation: no study should take off without verifying its scientific safety. When applied to neurology, especially in trials for neurodegenerative disorders, epilepsy, or neurorehabilitation, this approach can prevent wasted effort and improve the credibility of outcomes [31].

C. Strengthening collaboration and data openness: Global initiatives, such as the Human Connectome Project [33] and the UK Biobank [34], have shown the value of transparent data sharing. Expanding these models to include meta-data, information about how data were collected, analyzed, and reported, would allow researchers to assess not just results but the reliability of the processes behind them [28]. Journals and funding agencies can support this by rewarding openness and reproducibility as indicators of quality, not optional extras [28].

D. Integrating meta-research into evidence-based medicine education: Training future neurologists and neuroscientists should include not only how to read scientific papers but also how to question their methodological reliability. By embedding meta-research principles into medical curricula, residency programs, and continuing education, clinicians will be better equipped to identify weak evidence, interpret conflicting findings, and apply knowledge safely to patient care [35].

E. Translating meta-research into clinical policy and decision-making: Evidence-based neurology depends on the quality of the underlying studies. Health authorities, guideline developers, and professional societies should incorporate meta-research findings when creating recommendations or assessing new technologies [15,36]. A transparent evaluation of study quality could serve as a reliability filter, ensuring that only robust, reproducible evidence informs clinical decisions [15].

F. Building international networks for reflexive neuroscience: Finally, advancing meta-research requires global cooperation. Low- and middle-income countries, often underrepresented in neuroscience, should be actively included in international meta-research consortia [37]. This would not only democratize participation but also improve the external validity of neuroscientific evidence by representing diverse populations and research contexts [37].

It is important to note that the final sample size of 15 studies reflects the entire universe of peer-reviewed meta-research articles indexed in Scopus, as no temporal restrictions were applied and the search covered the full indexing history of the database. The limited number of records is therefore an intrinsic characteristic of the evidence available in the field, rather than a limitation of the study design. Preprints and gray literature were not included because the aim of this work was to characterize the maturity of formally published, peer-reviewed meta-research in neuroscience, which cannot be assessed through non-validated or non-reviewed sources. Then, temporal dynamics, inflection points, or emerging patterns cannot be robustly inferred. Future work may incorporate longitudinal approaches once meta-research in neuroscience expands and a larger, more continuous evidence base becomes available.

## 5. Conclusions

Meta-research in neuroscience is scarce, both in volume and scope, despite the enormous size of the field. Addressing this gap is imperative to ensure the validity, applicability, and equitable translation of neuroscientific evidence into clinical practice. Strengthening global collaborations, expanding participation beyond high-income countries, and fostering a culture of reflexivity are essential steps to advance neuroscience as a discipline capable of supporting truly evidence-based practice.

To facilitate progress, several concrete actions could be implemented across neuroscience subfields. First, preregistration of study designs and analysis plans can reduce selective reporting and analytical flexibility. Second, adopting standardized reporting checklists would improve transparency and methodological completeness. Third, promoting open data and open code practices through established repositories can enhance reproducibility and enable independent re-analysis. Fourth, institutions and research groups could incorporate routine methodological audits or meta-research assessments to identify recurrent sources of bias and guide improvements in research practices. Together, these measures provide practical pathways for strengthening the reliability, transparency, and clinical relevance of neuroscientific evidence.

## Data Availability

The original contributions presented in the study are included in the article. Further inquiries can be directed to the corresponding authors.
